# Hypophosphatemia in Coronavirus Disease 2019 (COVID-19), Complications, and Considerations: A Systematic Review

**DOI:** 10.1155/2022/1468786

**Published:** 2022-10-21

**Authors:** Mohammad Fakhrolmobasheri, Mehrbod Vakhshoori, Maryam Heidarpour, Arash Najimi, Amir Mohamad Mozafari, Hassan Rezvanian

**Affiliations:** ^1^Heart Failure Research Center, Cardiovascular Research Institute, Isfahan University of Medical Sciences, Isfahan, Iran; ^2^Isfahan Endocrine and Metabolism Research Center, Isfahan University of Medical Sciences, Isfahan, Iran; ^3^Medical Education Department, Medical Education Research Center, Education Development Center, Isfahan University of Medical Sciences, Isfahan, Iran; ^4^Health Information Technology Research Center, Clinical Informationist Research Group, Isfahan University of Medical Sciences, Isfahan, Iran

## Abstract

Coronavirus disease 2019 (COVID-19) has various manifestations on different body organs, including the lungs, heart, kidneys, and central nervous system. However, the frequency of electrolyte abnormalities, especially hypophosphatemia, is still debated in this pandemic. Our main aim in this review is to evaluate the frequency and complications of hypophosphatemia in COVID-19-infected individuals. A systematic literature review was performed in Web of Science, Scopus, PubMed, EMBASE, and Cochrane electronic databases with the combination of different keywords till October 2021. We recruited all relevant published records (including cross-sectional and case-control studies as well as editorials and brief reports) assessing hypophosphatemia among patients with COVID-19 infection. After assessing all 928 recruited records and discarding duplicates, 4 records met the inclusion criteria. Three articles were further included during a manual search of the literature. Overall, the included studies reported 1757 subjects (males: 51.3%), with the mean age ranging from 37.2 ± 13.6 years to 65.9 ± 13.9 years. Hypophosphatemia prevalence has been reported from 7.6% to 19.5%. Patients with the severe status of COVID-19 had a higher prevalence of low serum phosphate levels than those with moderate infection. This review indicates that hypophosphatemia might be categorized as a complication in clinical settings during the COVID-19 pandemic, requiring a high clinical suspicion to implement appropriate diagnostic and therapeutic interventions to prevent life-threatening outcomes. However, it needs to be more elucidated by further studies whether hypophosphatemia in severe COVID-19 is directly related to COVID-19 or is just a complication of severe illness.

## 1. Introduction

Coronavirus disease 2019 (COVID-19) is now a worldwide healthcare concern [1]. Although remarkable improvements in medical care, health promotion programs, and preventive strategies using modern technologies have been made since the emergence of this virus, the disease is still taking lives and negatively affecting individuals' mental health due to the disease itself or the lockdown and the consequent restrictions [2, 3]. COVID-19 is more than a simple respiratory viral infection. Cardiovascular, renal, hematologic, and metabolic disturbances have resulted in physicians' complicated course of management [4]. The situation may become more complicated in COVID-19 subjects with special baseline conditions, including renal failure and pregnancy, leading to considerable physiological alterations [5]. COVID-19 is associated with many abnormalities in vitamins, electrolytes, trace elements, and acid-base homeostasis [6–9]. Electrolyte abnormalities are a common complication observed in COVID-19 infection due to renal, gastrointestinal, metabolic, and adrenal disturbances [6, 10–13]. Among different electrolyte abnormalities, hypophosphatemia is a less considered phenomenon in which several organs might be affected and easily overlooked by the symptoms of the disease [14]. Hypophosphatemia, by itself, is a rare disorder mostly observed in hereditary conditions such as hereditary hypophosphatemic rickets. However, it may also be a complication of many diseases such as primary and secondary hyperparathyroidism, vitamin D deficiency, acute respiratory alkalosis, and refeeding syndrome [15]. Hypophosphatemia may occur in critically ill patients admitted to the intensive care unit (ICU) due to respiratory failure, massive burns, and renal disorders [16–18]. Hypophosphatemia could exacerbate the course of disease in such patients [19, 20]. It has been reported that up to 5% of patients admitted to hospitals might experience hypophosphatemia despite not showing clinical symptoms [21]. Serum phosphate levels lower than 2.5 mg/dl may manifest with various organ disturbances [21]. Several studies indicate that hypophosphatemia might be associated with an increased mortality rate among severely ill patients and delay the weaning time in intubated patients suffering from pneumonia [20]. Also, respiratory, cardiovascular, and hematologic disorders are common manifestations of deep hypophosphatemia [14]. Thus, it could be hard to distinguish the exact cause of these complications. However, the correction of hypophosphatemia might reverse the course of the disease. In this systematic review, we aimed to gather the available reports of hypophosphatemia in COVID-19 to better understand the probable pathophysiological mechanisms, incident, and subsequent management strategies.

## 2. Methods

This study was designed and reported according to the Preferred Reporting Items for Systematic Reviews and Meta-Analyses (PRISMA) protocol [22]. We also registered the current review in the International Prospective Register of Systematic Reviews (PROSPERO) database registry (CRD42021255908). It was conducted to review the available literature in terms of hypophosphatemia in COVID-19 patients. The study question was “what is the role, incidence, complications, and prognosis of hypophosphatemia in COVID-19 patients.” Therefore, the eligibility criteria for study inclusion were defined as any clinical observational human studies (including cross-sectional and case-control studies as well as editorials and brief reports) reporting the frequency, complications, and prognosis of hypophosphatemia in the COVID-19 pandemic.

**Figure 1:**
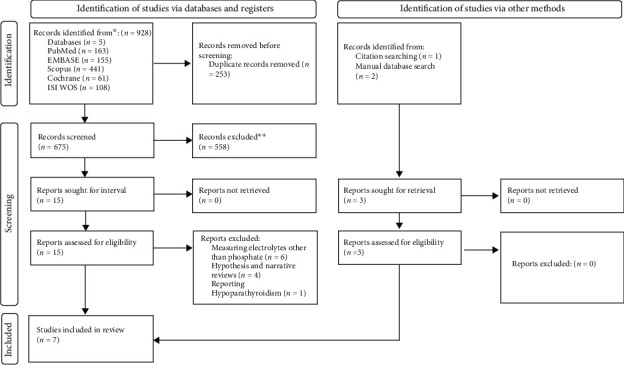
Flow diagram of the current study.

We conducted literature searching in different databases including Web of science, Scopus, PubMed, EMBASE, and Cochrane with the following items: (Hypophosphataemia OR hypophosphatemia OR hypophosphoremia OR fosfor OR phosphor OR phosphorus OR “inorganic orthophosphate” OR “inorganic phosphate” OR “inorganic phosphorus” OR orthophosphate OR phosphate^∗^ OR electrolyte^∗^) (in title) AND (“2019 novel coronavirus disease” OR “2019 novel coronavirus infection” OR “2019-nCoV disease” OR “2019-nCoV infection” OR COVID OR “COVID 19” OR “COVID 2019” OR “COVID-19” OR “COVID19” OR “SARS coronavirus 2 infection” OR “SARS-CoV-2 disease” OR “SARS-CoV-2 infection” OR “SARS-CoV2 disease” OR “SARS-CoV2 infection” OR “SARSCoV2 disease” OR “SARSCoV2 infection” OR “Wuhan coronavirus disease” OR “Wuhan coronavirus infection” OR “coronavirus disease 2019” OR “nCoV 2019 disease” OR “nCoV 2019 infection” OR “novel coronavirus 2019 disease” OR “novel coronavirus 2019 infection” OR “novel coronavirus disease 2019” OR “novel coronavirus infection 2019”) (in title, abstract) NOT Chloroquine (in title, abstract). We did not consider any time or language limitations during database searching. Case reports, case series, animal studies, or any records with incomplete data were excluded. Additionally, we manually reviewed the references of included studies. We updated our database search during the manuscript preparation process to consider any newly published article. The flow diagram of the study selection process is provided in Figure 1.

Two independent authors screened records, and relevant articles were selected for abstract and full-text review, as appropriate. Discrepancies were solved by consensus. In order to assess the risk of bias, we used the Appraisal tool for Cross-Sectional Studies (AXIS tool) [23] and Joanna Briggs Institute (JBI) critical appraisal tools, as appropriate [24]. The following items were extracted from eligible records: first author's name, design of the study, sample size, gender, age (mean ± standard deviation or median and interquartile range, as reported), phosphate levels, criteria for hypophosphatemia diagnosis, hypophosphatemia frequency, disease severity classification, and laboratory and clinical outcomes.

## 3. Results

### 3.1. Article Inclusion and Data Extraction

Our primary database search resulted in 928 records. After removing the duplicate items, 657 studies remained, and all records were screened regarding title relevancy. Sixteen articles were chosen for abstract review. Finally, four records on 438 patients were eligible, and their information was extracted. Moreover, during the article preparation period, we included 3 newly published articles (not published at the time of primary search) by manually reviewing the databases and references with a total of 1319 subjects, resulting in 7 articles with overall 1757 subjects.

### 3.2. Study Characteristics and Risk of Bias

Six out of the seven recruited studies had cross-sectional designs. The study by Pal et al. was designed as a case-control study [25]. The study by Chen et al. had the largest study population with 823 subjects and the smallest sample of 32 subjects in the study by Xue et al. [26, 27]. Four studies compared the serum phosphate mean between study groups [25, 27–29], and three studies reported the outcomes of COVID-19 and laboratory findings between patients with or without hypophosphatemia [26, 30, 31]. The studies by Chen et al. and Wang et al. defined hypophosphatemia as serum phosphate levels below 0.8 mmol/l. Yang et al. defined hypophosphatemia as serum phosphate levels below 0.85 mmol/l [26, 30, 31]. The summary of the risk of bias assessment of the recruited studies is shown in the supplementary appendix (Table S1 and S2).

### 3.3. Synthesis of Results

Regarding the heterogeneity of the populations and different patient classifications as well as measured outcomes, performing a meta-analysis was not possible.  Table 1 provides the detailed extracted information of recruited articles. Studies by Wang et al. and Chen et al. on 435 and 823 subjects, respectively, revealed that hypophosphatemia was associated with higher mortality rates in COVID-19-infected patients. Yang et al., in a study on 226 subjects, attempted to discover the ability of serum calcium and phosphorus for discriminative diagnosis of severe COVID-19. The authors reported a higher prevalence of hypophosphatemia in severe and critical COVID-19 patients. They indicated that lower serum phosphate levels correlate with liver impairment, lactic acidosis, serum creatine kinase, and C-reactive protein (CRP) [31].

In contrast, Pal et al., in a study comparing nonsevere COVID-19 patients with healthy controls, reported that phosphate did not correlate with CRP levels. However, they indicated that among nonsevere COVID-19 patients, there was a lower serum phosphate level than the normal population [25]. Javdani et al., in a study on 36 subjects, concluded that patients with the COVID-19 Reporting and Data System (CORADS) scores of V and IV had remarkably lower serum phosphate levels compared to those with CORADS scores of II and III. They also postulated that serum phosphate levels higher than 4.5 mg/dl were associated with better high-resolution computed tomography (HRCT) reports [29]. Xue et al. reported a high incidence (50%) of hypophosphatemia in severe and critical COVID-19 patients. They also suggested that phosphate levels might be correlated with absolute lymphocyte counts. Their results were in favor of the study by Yang et al., which indicated that combining serum phosphate levels with lymphocyte count could effectively help determine the prognosis of COVID-19. These findings might support the theory that hypophosphatemia could exacerbate the leukopenia in COVID-19 [27]. Arenas et al. performed a study on patients suffering concurrently from renal diseases and COVID-19 and found lower serum phosphate levels in confirmed COVID-19 patients than in suspected patients. They postulated that hypophosphatemia might result from malnutrition in these patients [28]. Chen et al. reported that hypophosphatemia is an independent risk factor for acute kidney injury (AKI). They also indicated that renal tubular dysfunction might be the cause of renal phosphate loss [26].

## 4. Discussion

**Figure 2:**
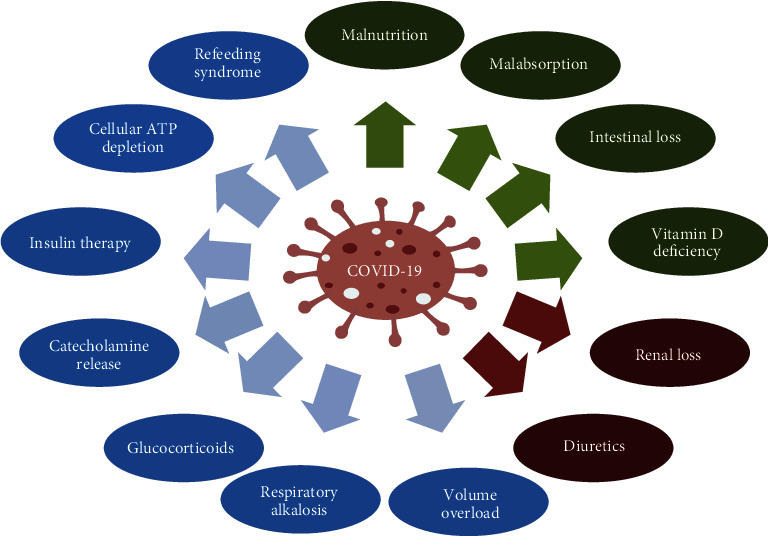
Potential risk factors associated with hypophosphatemia in COVID-19. Green color: associated with low intake and malabsorption. Vitamin D deficiency decreases intestinal and renal phosphate absorption. Malnutrition could be mostly secondary to chronic diseases, including end-stage renal disease, pulmonary diseases, or malignancy. Red color: associated with increased renal excretion of phosphate. Carbonic anhydrase inhibitors, thiazides, and furosemide are among the most common effective factors in hypophosphatemia induction. Proximal tubular damage in COVID-19 infection might be attributed to renal phosphate loss. Blue color: associated with cellular shift mechanism. Volume overload is mainly seen in the setting of heart failure. Respiratory alkalosis results in cellular glycolysis and further phosphate shift into the cells. Glucocorticoids reduce the renal reabsorption of phosphate in addition to the effects on glucose synthesis. Catecholamine release through induction of glycogenolysis could cause hypophosphatemia. Insulin therapy increases cellular phosphate demand to produce high-energy phosphate bonds, ultimately resulting in hypophosphatemia.

Dysregulation in serum phosphate levels in COVID-19 patients may be associated with several pathophysiological mechanisms [14]. Various clinical conditions, particularly in critically ill and intensive care unit (ICU) patients, are accompanied by low serum phosphorus. The etiology of hypophosphatemia is complex and multifactorial [15]. The classic classification of the etiology of hypophosphatemia is categorized as three main mechanisms that could play a role in the phosphate metabolism disturbances in COVID-19 (Figure 2).

### 4.1. Hypophosphatemia due to Inadequate Intake

Gastrointestinal involvement in COVID-19 is a common finding, usually manifesting as nausea, vomiting, and diarrhea, leading to water and electrolytes disturbances [32]. As hypothesized by Xue et al., intestinal mucosal inflammation may contribute to the malabsorption of macro- and micronutrients. Moreover, the complex interconnection between obesity and hypophosphatemia attracts special consideration as most hypophosphatemic patients with severe or critical COVID-19 were overweight male adults [27].

### 4.2. Hypophosphatemia due to Renal Loss

Renal impairment may cause both hypo- and hyperphosphatemia in patients. Although the prevalence of hyperphosphatemia in patients on dialysis is higher than hypophosphatemia, on the contrary, Arenas et al. reported a high incidence of hypophosphatemia in COVID-19 patients with concurrent end-stage renal diseases (ESRD). They postulated that this finding might be explained by severe malnutrition in ESRD patients with COVID-19 [28]. Other suggested mechanisms associated hypophosphatemia with renal dysfunction [26]. AKI may follow the course of COVID-19 through several mechanisms [13]. Prerenal AKI may occur due to severe dehydration and sequestration of water in the pulmonary interstitial tissue. However, the most common cause of renal impairment in patients infected with COVID-19 has been reported to be proximal tubulopathies, either vascular induced or by direct viral invasion, consequently resulting in electrolyte loss and further complications [33]. Chen et al. reported hypophosphatemia as an independent risk factor for AKI in COVID-19. They further indicated that renal proximal tubular dysfunction is a common finding in patients with renal dysfunction in COVID-19. They also reported the association between renal proximal tubular dysfunction and hypophosphatemia incidence [26]. Moreover, in a recent study on 41 COVID-19-infected patients without AKI or CKD, the authors reported a remarkable renal electrolyte loss related to renal tubular dysfunction [34].

On the other hand, the effect of COVID-19 on vitamin D, calcium, and phosphorus homeostasis is suggested to be associated with phosphate metabolism disturbances. Vitamin D depletion is common in severely ill COVID-19 patients [7]. The connection between abnormal kidney function, abnormal vitamin D metabolism, and hypophosphatemia has been demonstrated in a recent study done by Povaliaeva et al. [35]. The authors indicated that patients with severe COVID-19 had abnormal vitamin D metabolism, higher serum creatinine levels, and lower serum phosphate levels [35]. Several mechanisms have been proposed to explain the role of vitamin D in COVID-19. Regarding the immune-modulatory effect of vitamin D, the role in the function of the immune system during COVID-19 has been widely discussed [36]. However, the effects of vitamin D on calcium, magnesium, and phosphorus homeostasis are less investigated. Vitamin D deficiency may cause low serum calcium and phosphate levels due to renal loss and intestinal malabsorption [14]. In response to low serum calcium levels, the parathyroid hormone (PTH) increases the bone reabsorption, renal reabsorption, and intestinal absorption of calcium but concurrently increases renal phosphate loss [14]. In this regard, Pal et al. compared serum phosphorus levels in COVID-19 patients with age, sex, and vitamin D matched healthy controls. They found lower phosphorus levels among COVID-19 patients [25]. This finding contrasts with the previously proposed mechanisms for the role of vitamin D deficiency in phosphate metabolism disturbances in COVID-19 infection. However, more detailed investigations are needed to elucidate the interconnection between COVID-19, vitamin D, and phosphate metabolism.

### 4.3. Hypophosphatemia due to Cellular Shift

Xue et al. discussed the effect of stress response and severe systemic inflammation in COVID-19 patients as a contributor to hypophosphatemia [27]. Inflammation resulting from cytokine storms in COVID-19 poses a significant obstacle to normal cellular energy metabolism. Heavy oxidative stress combined with mitochondrial dysfunction leads to severe depletion of cellular adenosine triphosphate (ATP). The response to ATP depletion and impaired oxidative ATP synthesis results in upregulation of the glycolysis pathway requiring inorganic phosphors in the first step of enzymatic phosphorylation as well as increasing the cellular phosphate demand and the consequent hypophosphatemia. On the other hand, the insulin resistance caused by severe systemic inflammation provokes hyperinsulinemia which could also cause hypophosphatemia due to cellular shift [37].

Complications of hypophosphatemia are generally attributed to major consequences of cellular phosphate depletion as follows: the impairment in cellular ATP metabolism causes systemic tissue and organ dysfunction and increased affinity of hemoglobin to oxygen due to the fall in the 2,3-bisphosphoglycerate (2,3-BPG) concentration in red blood cells (RBC) which might exacerbate the cellular stress condition due to hypoxia [14]. In clinical settings, hypophosphatemia might be associated with neurologic dysfunction, respiratory and cardiovascular failure, renal failure, hematologic disorders, and musculoskeletal and smooth muscle dysfunctions [38].

In the context of COVID-19, hypophosphatemia could exacerbate the disease course through several mechanisms. The hypoxia due to pulmonary dysfunction accompanies 2,3-BPG depletion and cellular damage and triggers cardiac injury [11, 27]. The immune system dysfunction in COVID-19 is very complex and not fully understood. However, many abnormalities in trace elements, vitamins, and electrolytes are reported to correlate with immune system dysfunction in COVID-19 [27, 39, 40]. The role of hypophosphatemia in immune system dysfunction is a matter of debate. Studies indicate a positive relation of the CD4/CD8 ratio with serum phosphate levels in patients with pneumonia which might be consistent with the reduced CD4/CD8 ratio in COVID-19 [27]. In two separate studies, Heidarpour et al. [12] and Gaasbeek and Meinders [15] indicated the correlation between lower serum phosphate levels and leukocyte count in COVID-19 patients. Moreover, hypophosphatemia in COVID-19 patients may be a major contributor to other hematologic disturbances, including neutropenia [14].

Previous studies had associated the adverse outcomes of COVID-19 with dysregulated cellular and mitochondrial metabolism and proposed different targets for intervention to ameliorate the severe mitochondrial dysfunction in COVID-19 [41]. The effect of hypophosphatemia on energy metabolism in respiratory muscles might induce early respiratory failure, ultimately leading to intubation and further poor outcomes in intubated patients [14]. The direct impact of hypophosphatemia on lung tissue includes early cell apoptosis due to severe ATP depletion and possibly reduced surfactant secretion leading to acute respiratory distress syndrome (ARDS) [29]. Cardiovascular complications associated with COVID-19 result from complex myocardial and endothelial damage due to hypoxia, severe oxidative stress, drug intoxication, and viral invasion. Hypophosphatemia may worsen this situation by reducing the myocardial cellular ATP synthesis and endothelial dysfunction, contributing to cardiomyopathy and ischemia [38]. Renal injury, as a relatively common incident in severely ill COVID-19 patients, might be associated with proximal tubular dysfunction contributing to hypophosphatemia and be the consequence of hypophosphatemia in COVID-19 [33]. Although the neurologic manifestations of this virus are a subject of debate, it is clearly defined that hypoxia, in combination with endothelial dysfunction and blood-brain barrier dysfunctions, are major factors that occur in the context of hypophosphatemia. Thus, hypophosphatemia may be an aspect of the pathophysiology of neurologic disorders related to COVID-19 [15, 14, 19].

**Figure 3:**
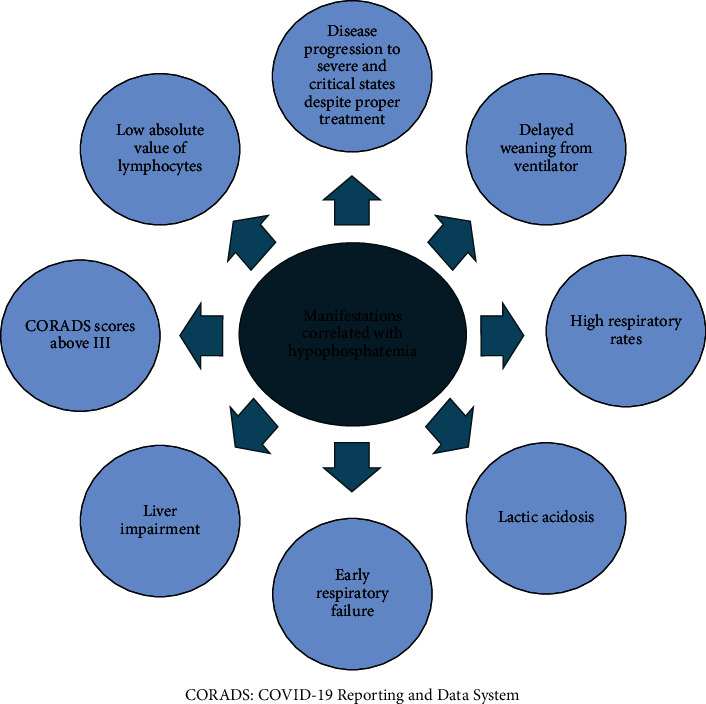
Manifestations correlated with hypophosphatemia.

Assuming COVID-19 as a systemic disease that mainly affects cellular metabolisms by severe oxidative stress and hypoxia, hypophosphatemia is a major complicit or imitator in many serious complications of the disease [42]. The manifestations reported correlating with hypophosphatemia during COVID-19 infection are provided in Figure 3.

Although there are heterogeneous reported results about the incidence and complications of hypophosphatemia in COVID-19, the fact that serum phosphate levels are negatively correlated with the severity of COVID-19 is elucidated. However, a recent study by Malinowska et al. stated that hyperphosphatemia could also contribute to the development of severe COVID-19. Their findings also indicated no significant association between hypophosphatemia and severity of COVID-19, but this finding should be considered cautiously regarding the small number of subjects (6% of the total population) [43]. Pal et al. indicated that COVID-19 patients had lower serum phosphate levels than the healthy population [25]. On the other hand, Javdani et al. postulated that phosphate levels higher than 4.5 mg/dl might be associated with better HRCT findings [29]. Moreover, Wang et al. [30] indicated that hypophosphatemia at admission is associated with worse outcomes in COVID-19 patients, and Chen et al.'s study postulated hypophosphatemia with the incidence of AKI in COVID-19 [26].

Taken altogether, it is clear that phosphate metabolism abnormalities in COVID-19 are associated with worse outcomes. It is also notable that in critically ill and ICU patients, regardless of the etiology, hypophosphatemia could occur, and different studies have evaluated the effect of phosphate supplementation in such patients. However, the results are inconsistent, and there is a need for further clinical trials [26, 30].

To the best of our knowledge, this study was the first in the literature assessed the frequency of hypophosphatemia in COVID-19-infected subjects. However, several limitations are still existing. We tried our best to include all relevant records in the current review. However, due to the daily basis of published records in the context of the COVID-19 entity, we just investigated records till October 2021. Although recruited records were in favor of the probable association between hypophosphatemia and severity of COVID-19, this issue is still a debate, and complementary studies are required to clarify this cause and effect relation. Moreover, we could not perform a meta-analysis due to the small number of recruited studies and high heterogeneity of recruited articles.

## 5. Conclusion

In conclusion, considering low phosphate levels as an indicator of severe disease in COVID-19 and a significant contributor to disease progression, serum phosphate measurement could be considered in clinical settings among patients suffering from COVID-19 infection.

## Figures and Tables

**Table 1:** Summary of recruited studies reporting hypophosphatemia in patients with COVID-19.

Authors	Date	Study design	Sample size			Age (years)	Male (%)	Levels of serum phosphate (mg/dl)	Hypophosphatemia (%)	Criteria for hypophosphatemia	Outcomes significantly correlating with serum phosphorus	Laboratory parameters significantly correlating with serum phosphorus
					*N* (%)							
Arenas et al. [28]	August 2020	Cross-sectional	Total		61 (100)	Mean ± SD: 65.9 ± 13.9	45 (73)	2.33 ± 1.96 vs. 4.50 ± 1.65	N/A	N/A	N/A	N/A
			ESRD patients, confirmed for COVID-19		34 (55.7)	Mean ± SD: 69 ± 10.1	24 (70.6)					
			ESRD patients, suspected of COVID-19		27 (44.3)	Mean ± SD: 62.1 ± 17	21 (77.8)					

Chen et al. [26]	September 2021	Cross-sectional	Total		823 (100)	Mean ± SD: 60.9 ± 14.9	412 (50.1)	3.38 (2.91-3.78)	60/637 (9.4)	Serum phosphate under 0.8 mmol/l (2.48 mg/dl)	AKI, death, longer hospitalization	Lower lymphocyte count, serum albumin, acid uric, higher LDH and CRP
			Acute kidney injury		44 (6)	Mean ± SD: 72 ± 11.4	27 (61.4)	2.51 (1.98-3.34)				
			No acute kidney injury		779 (94)	Mean ± SD: 60.2 ± 14.9	385 (49.4)	3.41 (2.94-3.78)				

Javdani et al. [29]	August 2020	Cross-sectional	Total		36 (100)	<30 years: 2 (5.6) 30-60 years: 18 (50) ≥60 years:16 (44.4)	20 (55.6)	5.36 ± 1.6 vs. 3.43 ± 1.15	N/A	N/A	N/A	N/A
			CORAD II and III		5 (13.8)	N/A	N/A					
			CORAD IV and V		31 (86.2)	N/A	N/A					

Pal et al. [25]	January 2021	Case-control	Total		144 (100)	Mean ± SD: 37.2 ± 13.6	68 (47)	3.3 ± 0.7 vs. 3.5 ± 0.5	N/A	N/A	N/A	N/A
			Nonsevere		72 (50)	Median (IQR): 36 (27-48.2) Mean ± SD: 37.5 ± 13.7	34 (48)					
			Healthy controls		72 (50)	Median (IQR): 35 (25.2-45.2) Mean ± SD: 36.9 ± 13.7	34 (48)					

Wang et al. [30]	September 2021	Cross-sectional	Total		435 (100)	57 (41-68)	200 (46)	3.44 (2.97-3.87)	33 (7.6)	Serum phosphate under 0.8 mmol/l (2.48 mg/dl)	Mortality	Lower lymphocyte count
			Nonhypophosphatemia		402 (92.4)	56 (39-67)	187 (46.5)	3.5 (3.07-3.96)				
			Hypophosphatemia		33 (7.6)	67 (63-76)	13 (39.4)	2.26 ()				

Xue et al. [27]	March 2020	Cross-sectional	Total		32 (100)	Mean ± SD: 48.2 ± 4.5	20 (62.5)	3.44 ± 1.08 vs. 2.45 ± 0.09	N/A	N/A	N/A	Absolute value of lymphocyte
			Severe		20 (62.5)	Mean ± SD: 49.1 ± 3.9	12 (60)		10 (50)			
			Ordinary		12 (37.5)	Mean ± SD: 46.7 ± 5.3	8 (66.7)		N/A			

Yang et al. [31]	September 2020	Cross-sectional	Total		226 (100)	Mean ± SD: 40.6 ± 19.3 Median (IQR): 40 (28-54)	137 (60.6)	N/A	44 (19.5)	Serum phosphate under 0.85 mmol/l (2.65 mg/dl)	ICU admission, ICU stay, CT score, oxygen saturation, respiratory rate	ALT/AST, CRP, lactic acid, CK, Ca
			Suspected COVID-19		122 (54)	Median (IQR): 35 (26-53)	73 (59.8)		13 (10.9)			
			Confirmed COVID-19	Total	104 (46)	Median (IQR): 44 (33-55)	64 (61.5)		30 (29.7)			
				Severe confirmed	36 (35)	Median (IQR): 47 (41-64)	24 (66.6)		20 (57.1)			
				Moderate confirmed	68 (65)	Median (IQR): 42 (33-52)	39 (58.8)		10 (15.2)			

N/A: not available; IQR: interquartile range; SD: standard deviation; COVID-19: coronavirus disease 2019; ESRD: end-stage renal diseases; CORAD: COVID-19 Reporting and Data System; ICU: intensive care unit; CT: computed tomography; ALT: alanine aminotransferase; AST: aspartate aminotransferase; CRP: C-reactive protein; CK: creatine kinase; Ca: calcium.

## Data Availability

The datasets generated during and/or analyzed during the current study are not publicly available due to confidential issues but are available from the corresponding author on reasonable request.

## Abbreviations

COVID-19: Coronavirus disease 2019
PRISMA: Preferred Reporting Items for Systematic Reviews and Meta-Analyses
PROSPERO: Prospective Register of Systematic Reviews
AMSTAR: Assessment of multiple systematic reviews
STROBE: Strengthening the Reporting of Observational Studies in Epidemiology
CRP: C-reactive protein
CORADS: COVID-19 Reporting and Data System
HRCT: High-resolution computed tomography
ESRD: End-stage renal diseases
AKI: Acute kidney injury
PTH: Parathyroid hormone
ATP: Adenosine triphosphate
2,3-BPG: 2,3-Bisphosphoglycerate
RBC: Red blood cell
ARDS: Acute respiratory distress syndrome.

## Supplementary Materials

Supplementary Materials Table 1 shows the results of risk of bias assessment of recruited cross-sectional studies. Table 2 presents the results of risk of bias assessment of recruited case-control study.
