# Supplementary material for: Hypophosphatemia in Coronavirus Disease 2019 (COVID-19), Complications, and Considerations: A Systematic Review
Source: Biomed Res Int. 2022 Oct 21;2022:1468786. doi: 10.1155/2022/1468786 (PMC9616661; doi:10.1155/2022/1468786)
Supplement: Supplementary Materials — Table 1 shows the results of risk of bias assessment of recruited cross-sectional studies. Table 2 presents the results of risk of bias assessment of recruited case-control study. [file 1468786.f1.docx]

**Supplementary materials**

**Table S1. Risk of bias assessment of included studies with the cross-sectional design using AXIS tool**

| Components | Yang  et al.. | Chen et al. | | Xue et al. | | Javdani  et al. | | Arenas et al. | |  | | Wang et al. | |
| --- | --- | --- | --- | --- | --- | --- | --- | --- | --- | --- | --- | --- | --- |
| 1. Were the aims/objectives of the study clear? | Yes | Yes | Yes | | Yes | | Yes | |  | | Yes | |  |
| 2. Was the study design appropriate for the stated aim(s)? | Yes | Yes | Yes | | Yes | | Yes | |  | | Yes | |  |
| 3. Was the sample size justified? | No |  | No | | No | | No | |  | | No | |  |
| 4. Was the target/reference population clearly defined? (Is it clear who the research was about?) | Yes | Yes | Yes | | Yes | | Yes | |  | | Yes | |  |
| 5. Was the sample frame taken from an appropriate population base so that it closely represented the target/reference population under investigation? | Yes | Yes | Yes | | Yes | | Yes | |  | | Yes | |  |
| 6. Was the selection process likely to select subjects/participants that were representative of the target/reference population under investigation? | Yes | Yes | Yes | | Yes | | Yes | |  | | Yes | |  |
| 7. Were measures undertaken to address and categorize non-responders? | No | No | No | | No | | No | |  | | Yes | |  |
| 8. Were the risk factor and outcome variables measured appropriate to the aims of the study? | Yes | Yes | Yes | | Yes | | Yes | |  | | Yes | |  |
| 9. Were the risk factor and outcome variables measured correctly using instruments/ measurements that had been trialed, piloted, or published previously? | Yes | Yes | Yes | | Yes | | Yes | |  | | Yes | |  |
| 10. Is it clear what was used to determine statistical significance and/or precision estimates? (e.g., p values, CIs) | Yes | Yes | Yes | | Yes | | Yes | |  | | Yes | |  |
| 11. Were the methods (including statistical methods) sufficiently described to enable them to be repeated? | Yes | Yes | Yes | | Yes | | Yes | |  | | Yes | |  |
| 12. Were the basic data adequately described? | Yes | Yes | Yes | | Yes | | Yes | |  | | Yes | |  |
| 13. Does the response rate raise concerns about non-response bias? | No | No | No | | No | | No | |  | | No | |  |
| 14. If appropriate, was information about non-responders described? | No | No | No | | No | | No | |  | | Yes | |  |
| 15. Were the results internally consistent? | Yes | Yes | Yes | | Yes | | Yes | |  | | Yes | |  |
| 16. Were the results for the analyses described in the methods presented? | Yes | Yes | Yes | | Yes | | Yes | |  | | Yes | |  |
| 17. Were the authors’ discussions and conclusions justified by the results? | Yes | Yes | Yes | | Yes | | Yes | |  | | Yes | |  |
| 18. Were the limitations of the study discussed? | Yes | Yes | Yes | | Yes | | Yes | |  | | Yes | |  |
| 19. Were there any funding sources or conflicts of interest that may affect the authors’  interpretation of the results? | No | No | No | | No | | No | |  | | No | |  |
| 20. Was ethical approval or consent of participants attained? | Yes | Yes | Yes | | Yes | | No | |  | | No | |  |

**Table S2. Risk of bias assessment of included studies with the case-control design using JBI's critical appraisal tool**

| **Components** | **Pal et al.** |
| --- | --- |
| 1. **Were the groups comparable other than the presence of disease in cases of the absence of disease in controls?** | Yes |
| 1. **Were cases and controls matched appropriately?** | Yes |
| 1. **Were the same criteria used for the identification of cases and controls?** | Yes |
| 1. **Was exposure measured in a standard, valid and reliable way?** | Yes |
| 1. **Was exposure measured in the same way for cases and controls?** | Unclear |
| 1. **Were confounding factors identified?** | No |
| 1. **Were strategies to deal with confounding factors stated?** | No |
| 1. **Were outcomes assessed in a standard, valid and reliable way for cases and controls?** | Yes |
| 1. **Was the exposure period of interest long enough to be meaningful?** | Not applicable |
| 1. **Was appropriate statistical analysis used?** | Yes |
